# Imaging and histopathologic characteristics of typical pancreatic hamartoma: a case report and literature review

**DOI:** 10.3389/fonc.2024.1418244

**Published:** 2024-08-19

**Authors:** Shunli Liu, Lei Yang, Jie Wu, Xin Lin, Zaixian Zhang

**Affiliations:** ^1^ Department of Radiology, The Affiliated Hospital of Qingdao University, Qingdao, China; ^2^ Department of Radiology, Qingdao Women and Children’s Hospital, Qingdao, China; ^3^ Department of Pathology, The Affiliated Hospital of Qingdao University, Qingdao, China

**Keywords:** pancreatic hamartoma, computed tomography, magnetic resonance imaging, immunohistochemistry, imaging - radiology

## Abstract

**Background:**

Pancreatic hamartoma, a rare benign non-neoplastic condition, presents challenges in differentiating from other pancreatic diseases due to its atypical imaging and unreliable biopsy results. In this study, we present a case of pancreatic hamartoma and conduct a comprehensive review of relevant literature to outline its characteristic features, aiming to underscore its clinical relevance and implications.

**Case presentation:**

A 63-year-old man presented with a pancreatic mass, discovered during evaluation of abdominal pain and distension. Laboratory tests were largely unremarkable. Ultrasound revealed a hypoechoic mass in the head of the pancreas. Subsequent computed tomography and magnetic resonance imaging demonstrated an inhomogeneous mass with a clear boundary in the uncinate process of the pancreas. Furthermore, a distinct delayed enhancement pattern was noted on imaging. Histopathological examination confirmed the diagnosis of pancreatic hamartoma.

**Conclusions:**

Preoperative diagnosis of pancreatic hamartoma remains challenging. Imaging modalities can play a crucial role in facilitating accurate diagnosis and potentially avoiding unnecessary surgical intervention in patients with this condition.

## Introduction

Pancreatic hamartomas (PHs) are benign, non-neoplastic lesions arising from abnormal arrangement and combination of mature tissues and cells within the pancreas. These lesions are exceedingly rare, accounting for less than 1% of pancreatic tumor-like conditions (1). To date, fewer than 50 cases of pancreatic hamartoma have been documented. The mean age of these cases, which includes 4 pediatric cases, was 52 years (ranging from 34 weeks to 78 years), and no gender predominance was observed (2). Given the uncertainty surrounding their pathogenesis and the absence of characteristic imagining features, PHs are frequently misdiagnosed as other pancreatic tumors, potentially leading to inappropriate treatments or unnecessary interventions. Therefore, understanding the distinctive imaging features and histopathological characteristics that differentiate PH from other pancreatic pathologies is crucial for accurate diagnosis and optimal patient management. Herein, we present a case of pancreatic hamartoma and provide a review of the clinical, pathological, and imagining findings reported in the relevant literature.

## Case presentation

The case report was approved by our institutional review board, which waived the requirement for obtaining informed consent from patient.

**Figure 1:**
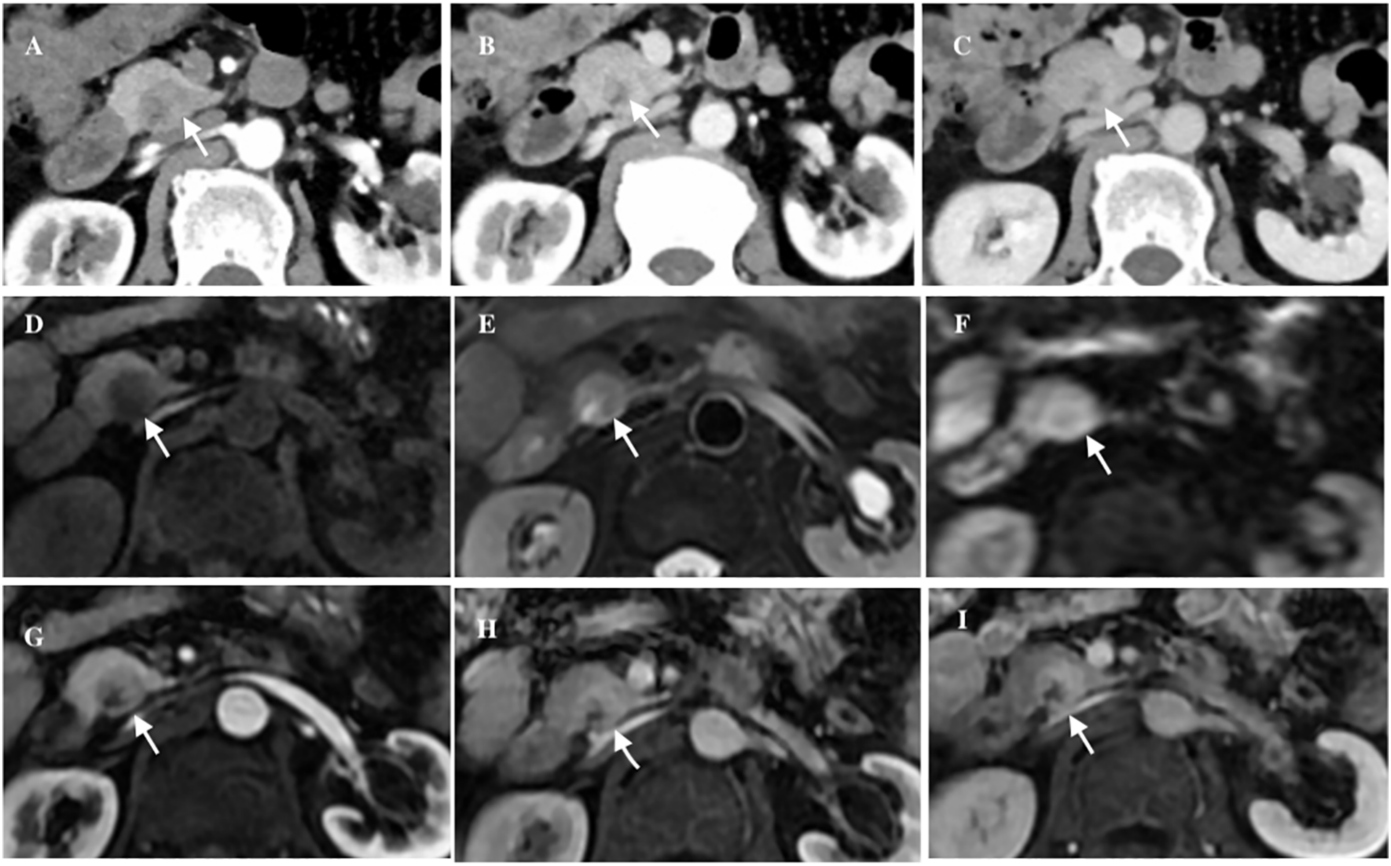
The axial abdominal CT enhanced scan revealed an inhomogeneous low-density lesion located in the uncinate process of the pancreas (white arrow), exhibiting a distinct boundary, slight enhancement in the arterial phase **(A)**, obvious enhancement in the venous phase **(B)** and iso- to hyper-density in the delayed phase **(C)**. Axial abdominal MRI plain scan showed a cystic and solid mass (white arrow), low intensity in T1WI **(D)**, iso- to high-intensity in T2WI and DWI **(E, F)**, and patchy cystic area could be seen inside. Axial abdominal MR enhanced scan showed the lesion (white arrow) had obvious progressive enhancement **(G-I)**.

A 63-year-old male patient presented to our hospital with a chief complaint of ‘abdominal distension with abdominal pain for half a month’. He had no history of pancreatitis or systemic diseases, and additionally, he underwent right inguinal hernia repair surgery over 10 years ago. Laboratory investigations revealed unremarkable results, including normal levels of carbohydrate antigen 19-9 (CA19-9), carbohydrate antigen 12-5 (CA12-5), carcinoembryonic antigen (CEA), immunoglobulin G4 (IgG4), and amylase. Ultrasonography revealed a hypoechoic mass in the head of the pancreas with a clear boundary. CT imaging further demonstrated an inhomogeneous low-density mass located in the uncinate process of the pancreas, measuring approximately 21 × 18mm^2^ in size, and also exhibiting a distinct boundary. Post-contrast CT scans showed slight enhancement of the lesion in the arterial phase, followed by more pronounced enhancement in both the venous and delayed phases. Additionally, patchy areas of non-enhancement were observed within the lesion (**Figure 1**). No evidence of fat was detected within the lesions on imaging. Magnetic resonance imaging (MRI) further confirmed the presence of a space-occupying lesion in the uncinate process of the pancreas, which appeared to be disconnected from the main pancreatic duct. On T1-weighted images (T1WI), the lesion exhibited low-intensity characteristics, while on T2-weighted images (T2WI) and diffusion-weighted images (DWI), it demonstrated iso- to high-intensity signals. Contrast-enhanced MRI revealed a distinct delayed enhancement pattern of the lesion compared to the surrounding pancreatic parenchyma. Given these findings, we included neuroendocrine tumors (NETs) in our differential diagnosis. However, NETs did not correlate with all radiological findings. It is impossible to definitively rule out malignancy. Given the increased likelihood of malignancy or premalignancy in pancreatic incidentalomas, surgical intervention is the recommended treatment.

**Figure 2:**
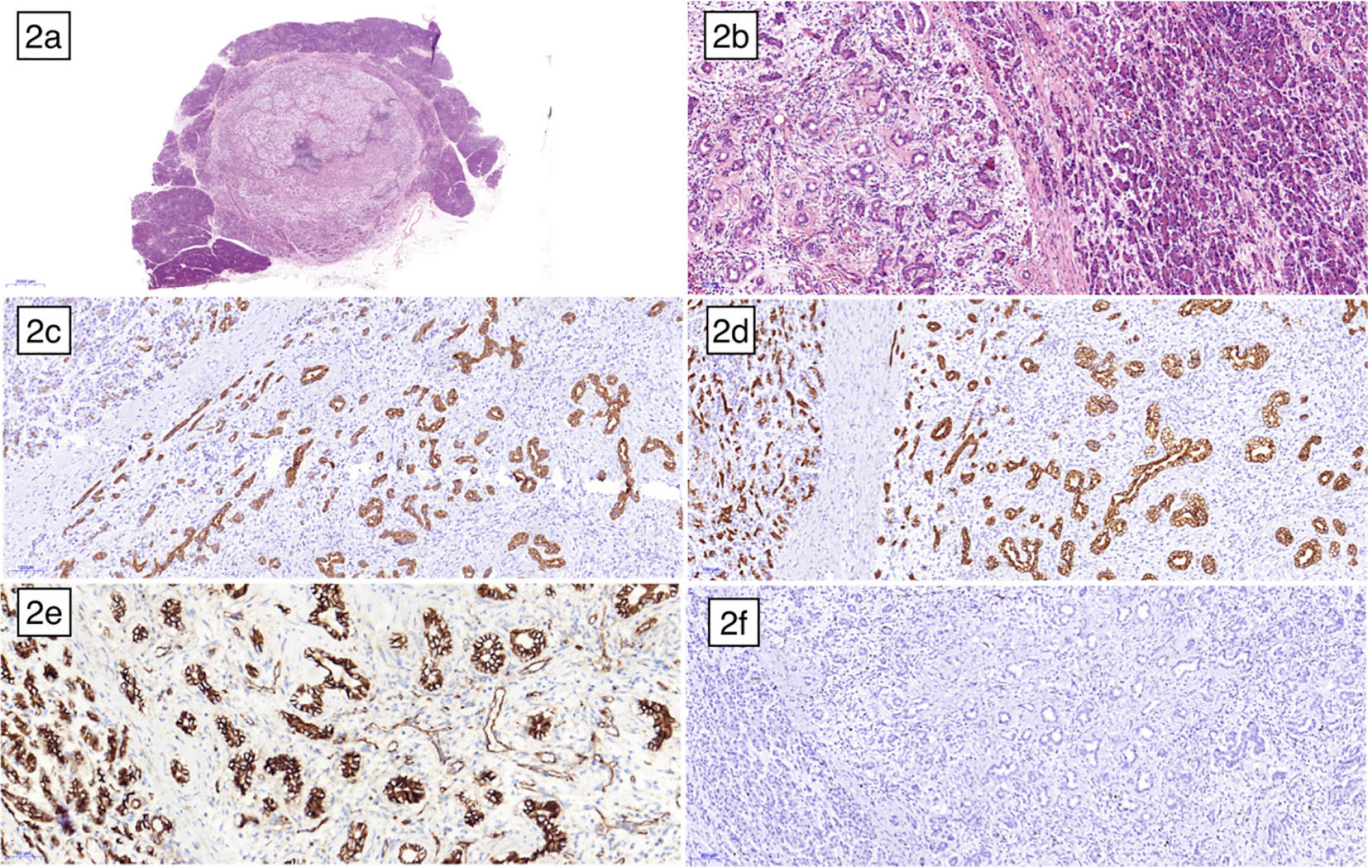
Microscopic examination under low magnification **(A)** and high magnification **(B)** revealed a distinct boundary between the pancreatic hamartoma and the normal pancreas, with no true capsule. The lesion was composed of disorganized ductal cells and acinar cells embedded within a delicate fibrous stroma. Immunohistochemical staining showed CK7 **(C)**, CK19 **(D)** and β-catenin membrane **(E)** positive, respectively. **(F)** illustrated the low expression of ki67.

One week later, the patient underwent a pancreaticoduodenectomy. Macroscopic examination of the surgical specimen revealed a firm, well-encapsulated lesion with a distinct boundary. Microscopic examination showed the lesion to be composed of well-differentiated pancreatic acini and disorganized ducts, surrounded by a fibrotic stroma (**Figure 2**). Immunohistochemistry analysis revealed positive staining for CK7, CK19, and β-catenin membrane, while CD56, CgA, and Syn were negative. The Ki-67 proliferation index was less than 5%. Based on these findings, the lesion was pathologically diagnosed as a pancreatic hamartoma. The patient was followed up for 16 months after surgery, during which time they recovered well with no signs of recurrence.

## Discussion

PH is an exceptionally rare, benign lesion characterized by tissue dysplasia. It was first described by Anthony et al. in 1977 (3). A query of the PubMed database utilizing the keyword words “pancreatic hamartoma” yielded 50 cases, predominantly in the form of case reports, including the current case. A review of the clinical, pathological, and imaging features of these cases is presented (1–38). **Table 1** summarized the relevant clinical and histopathological data of the 31 reported cases of PHs. The occurrence of PH has been reported in individuals ranging from neonates (21) to the elderly (14, 28). Notably, there is no significant gender predilection for PH. In cases where the lesion is small, patients may remain asymptomatic. However, larger lesions can manifest with a range of symptoms and signs, including abdominal pain, palpable abdominal mass, and weight loss. Notably, only one reported case presented with jaundice due to common bile duct obstruction (24). Serum tumor marker levels (e.g., CA19-9, CA12-5, CEA) are typically within normal limits or mildly elevated in patients with PH.

**Table 1:** Clinical-pathological characteristics of pancreatic hamartomas described in the literature (n = 32).

Author	Year	Age	Sex	Symptom	Tumor markers	Location	Size (mm)	Pathologic type
Addeo et al. (25)	2014	61y	F	Asymptomatic	CA19-9, CEA, CA125(Normal)	Body	26	S
Durczynski et al. (29)	2011	69y	M	Asymptomatic	CA19-9, CEA(Normal)	Body	28	S
Han et al. (19)	2018	35y	F	Hypoglycemia	CA19-9, CEA(Normal)	Tail	10	S
Tanaka et al. (18)	2018	54y	M	Asymptomatic	–	Body	36	S contains fat
Tanaka et al. (18)	2018	74y	M	Asymptomatic	–	Head	50	S contains fat
Tanaka et al. (18)	2018	67y	M	Asymptomatic	–	Tail	65	S/C contains fat
Inoue et al. (24)	2014	65y	M	Jaundice	CA19-9(Elevated)	Head	40	S
Kawakami et al. (28)	2012	78y	F	Asymptomatic	CA19-9, CEA(Normal)	Head	18	S
Kim et al. (1)	2012	52y	F	abdominal discomfort	CA19-9(Normal), CEA(Elevated)	Head	20	S/C
Matsushita et al. (23)	2016	68y	M	Asymptomatic	CA19-9, CEA(Normal)	Head	42	S/C, contains fat
Nagano et al. (20)	2017	72y	F	Asymptomatic	CA19-9, CEA(Normal)	Head	20	S
Zhang et al. (22)	2016	53y	F	Abdominal pain, weight loss	CA19-9, CA125, CA72-4(Normal)	Head	22	S
Izbicki et al. (34)	1994	25y	M	Abdominal pain, nausea	CEA(Elevated)	Head	106	S/C
Sampelean et al. (30)	2009	46y	M	Abdominal pain, weight loss	CA19-9, CEA(Normal)	Head	90	S/C
Nagata et al. (31)	2007	58y	F	Asymptomatic	(Normal)	Body	20	S
Pauser et al. (38)	2005	36y	F	Abdominal pain	–	Head	70	S/C
Pauser et al. (38)	2005	55y	F	Abdominal pain	–	Neck	30	S/C
Delgado et al. (21)	2017	33w	F	Asymptomatic	–	Diffuse	12	–
Noguchi et al. (9)	2021	70y	F	Asymptomatic	CA19-9, CEA(Normal)	Body	18	S
Ahn et al. (10)	2021	68y	M	Asymptomatic	CA19-9, CEA(Normal)	Head	18	S
Santana et al. (8)	2021	41y	M	Asymptomatic	CA19-9, CEA(Normal)	Body	19	S
Woo et al. (2)	2022	49y	F	Asymptomatic	CA19-9, CEA(Normal)	Body	10	S
Kim et al. (5)	2023	57y	F	Asymptomatic	–	Head	33	S
Shintaku et al. (4)	2023	68y	F	Asymptomatic	–	Head	18	S
Zhou et al. (11)	2020	73y	M	Abdominal pain	CA19-9, CEA(Normal)	Head	41	S, contains fat
Cui et al. (15)	2020	57y	F	Asymptomatic	–	Head	29	S
Cui et al. (15)	2020	69y	M	Asymptomatic	–	Head	15	S
Toyama et al. (12)	2020	53y	M	Asymptomatic	CA19-9, CEA(Normal)	Head	35	S/C
Katayama et al. (14)	2020	78y	M	Asymptomatic	CA19-9, CEA(Normal)	Tail	22	S
Nahm et al. (17)	2019	42y	F	Abdominal pain	CA19-9, CEA(Normal)	Neck	28	S
Shin et al. (16)	2019	54y	F	Asymptomatic	CA19-9, CEA(Normal)	Head	22	S
The present case	2023	63y	M	Abdominal pain	CA19-9, CEA(Normal)	Head	21	S

F, female; M, male; y, years; w, weeks; -, no reported; S, solid; S/C, solid and cystic.

Histologically, PHs are characterized by the presence of well-differentiated pancreatic acinar cells, ductal cells, and islet cells in varying proportions (31). Pathologically, PHs can exhibit two distinct patterns: solid and cystic solid. Solid PHs are primarily composed of fibrous and adipose tissue, while cystic solid PHs contain dilated pancreatic ducts, in addition to the solid components. Microscopic examination of the resected specimen in our case revealed lobulated arrangements of acinar cells and ductal structures within the hamartomatous tissue, resembling islet-like formations. However, no distinct islet cells were identified. In other reported cases of PH, the presence of islet cells has been a subject of debate, with conflicting evidence regarding their existence (27, 31). The histopathological findings highlight the significance of immunohistochemical markers used in distinguishing pancreatic hamartomas (PH) from other lesions. In **Figures 2C, D**, pancreatic ducts and duct-like structures showed positivity for cytokeratins CK7 and CK19, whereas normal pancreatic acinar cells were negative for these markers. CK7 and CK19 are typically expressed in ductal epithelial cells, contributing to the differentiation of PH from other lesions with distinct expression patterns. **Figure 2E** demonstrated that both normal pancreatic cells and hamartoma cells exhibited cell membrane and cytoplasmic positivity for β-Catenin, but nuclear staining was negative. In PH, β-Catenin typically exhibits membrane-bound positivity, contrasting with nuclear accumulation observed in other lesions such as solid pseudopapillary tumors. Neuroendocrine markers like CD56, Chromogranin A (CgA), and Synaptophysin (Syn) are usually negative in PH, aiding in its distinction from neuroendocrine tumors despite potential histological similarities. In our case, PH cells were negative for these markers, consistent with the absence of pancreatic islet cells, which typically stain positive for them.

Histopathologically, PHs exhibit similarities to both normal pancreatic tissue and chronic pancreatitis. The primary diagnostic criterion for PH on histopathology is the absence of specific normal pancreatic components, including concentric elastic fibers surrounding the ductal wall, peripheral nerves, and well-formed Langerhans islets (17). Fine-needle aspiration or biopsy of PHs may yield limited tissue, potentially showing only normal ductal cells. This limited sampling can hinder accurate diagnosis (28). However, the role of endoscopic ultrasound biopsy has evolved significantly with the availability of new-generation needles and the incorporation of immunohistochemistry. These advancements enable the diagnosis of even rare diseases and facilitate appropriate patient management (39).

The most common location of PHs is the pancreatic head, although they can also occur in the body and tail of the pancreas. Existing reports indicate that imaging results lack specificity. Commonly, CT scans reveal a distinct mass exhibiting either isodense or hypodense properties. On occasion, fat density (7, 18, 23) or calcifications (32) may be visualized, albeit rarely.

MRI typically shows low signal intensity on T1WI and isointense to hyperintense signal intensity on T2WI and DWI (2, 9, 10, 12, 15, 19, 20, 23). Furthermore, the internal signal intensity of the lesions may be slightly heterogeneous and frequently accompanied by cystic changes. Dilation of the main pancreatic duct (MPD) and common bile duct is uncommon. However, larger lesions in the proximal pancreas can lead to pancreatic duct compression and subsequent dilation of the distal pancreatic duct (2, 20, 24, 25). A distinctive feature of PHs on MRI is the presence of progressive enhancement in the delayed phase (2, 9–15, 19, 20, 31). This finding is attributed to the increased fibrous components within the lesions. Histopathological examination of the less-enhancing regions within the solid components has revealed the presence of edematous fibrous interstitium (4, 10–12). The inhomogeneous delayed enhancement of the solid components within the lesions may serve as an imaging feature indicative of pancreatic hamartoma. The diagnostic value of 18F-FDG PET in PH remains uncertain. Most studies have reported normal or low FDG uptake in lesions (12–14, 16, 19), with high FDG uptake being uncommon (20). PH should be differentiated from pancreatic cancer, neuroendocrine tumors (NETs) and solid pseudopapillary tumors (SPTs) based on imaging findings. Pancreatic cancer typically manifests with delayed low enhancement and invasion of surrounding tissues (40, 41). Additionally, it often involves concurrent atrophy of the pancreatic body and tail, along with dilation of the primary pancreatic duct, thereby increasing propensity for local structural infiltration and distal metastasis. While most pancreatic NETs exhibit a rich blood supply, some high-grade NETs demonstrate mild enhancement in the arterial phase, followed by increased enhancement in the venous and delayed phases. This similarity in enhancement patterns can make it challenging to differentiate these tumors from pancreatic hamartoma on imaging. SPTs of the pancreas typically occur in young women and often present as cystic and solid masses. On enhanced imaging, SPTs may exhibit the characteristic “floating cloud sign” along with mild to moderate delayed enhancement. This comparison aims to underscore the challenges in preoperative diagnosis and highlight the distinct radiological features that differentiate PH from these entities.

Management of PH typically involves surgical resection, which is curative in most cases due to the lesion’s benign nature. However, due to the potential for recurrence or residual disease, postoperative follow-up is crucial. Regular clinical evaluations and imaging studies such as CT or MRI are essential to monitor for recurrence, although it is rare. These measures facilitate early detection and intervention if necessary, thereby optimizing patient outcomes following surgery.

## Conclusion

To summarize, the low incidence of pancreatic hamartoma (PH) and its lack of characteristic imaging findings make preoperative diagnosis challenging. PH should be considered in the differential diagnosis of pancreatic masses to distinguish them from benign and malignant lesions. Imaging is crucial for accurate diagnosis and prevention of misdiagnosis. Patient and healthcare provider education on the importance of follow-up visits and symptom awareness enables early detection of recurrence or complications, enhancing patient outcomes. To date, no recurrences or metastases have been reported in the literature. Our patient also experienced a favorable outcome following pancreaticoduodenectomy, with no evidence of recurrence after 20 months of follow-up.

## Data Availability

The raw data supporting the conclusions of this article will be made available by the authors, without undue reservation.
